# Legal and Ethical Issues Regarding Minors in the Italian Coronavirus Flu Emergency

**DOI:** 10.3389/fped.2021.544461

**Published:** 2021-01-26

**Authors:** Piergiorgio Fedeli, Nunzia Cannovo, Roberto Scendoni, Mariano Cingolani

**Affiliations:** ^1^School of Law, Legal Medicine, Camerino University, Camerino, Italy; ^2^Federico II University Ethics Committee, Naples, Italy; ^3^Law Department, Legal Medicine Section, Macerata University, Macerata, Italy

**Keywords:** children, COVID-19, Italian law, school, care

## Abstract

On February 21, 2020, Italy became one of the countries hit by an epidemic of the new coronavirus that causes “severe acute respiratory syndrome coronavirus 2” (SARS-CoV-2). Even a month before that, however, the Italian government began issuing a series of decrees and ordinances aimed at the containment of the virus in Italy, the first of them on January 25, 2020. The COVID 19 infection has been faced as an epidemic through measures to enforce a high degree of isolation. These regulations hold for minors, as well, with consequent difficulties for this age group. While at the moment young people appear to be the least vulnerable to the severe complications of COVID 19, the psychological problems that may be brought on by pandemic-related restrictions should be taken into serious consideration.

## Introduction

In 2017 in Italy, researchers identified a coronavirus cluster in bats in northern Italy, with peculiar genetic characteristics (1), but no further inquiry was conducted on its virulence in the hosts. Apparently, that viral cluster differed from the one isolated in the population of the Wuhan region of China, which then spread to 17 other countries (2).

The new coronavirus causes “severe acute respiratory syndrome coronavirus 2” (SARS-CoV-2), so named because it is correlated to the coronavirus that provoked SARS (SARS-CoVs). SARS-CoV-2 is classified genetically within the Betacoronavirus Sarbecovirus subgenus. The estimate of the SARS-CoV-2 basic reproduction number (R_O_), which describes the intensity of an infectious disease outbreak, varies from the WHO figure of 1.4–2.5 (3) to the University of York calculation of 6.47 (4).

The main source of contagion is sick patients or those who are in the incubation period of the virus (5). Propagation happens person-to-person through respiratory droplets transmitted when a patient sneezes or coughs (6). Currently, there is no demonstration of vertical transmission or transmission through maternal milk.

At greatest risk of grave pulmonary and cardiac complications (diffuse alveolar damage, vascular thrombosis, myocardial infarction) (7) are those with chronic diseases and low immune response (8), including the elderly, while the symptomatology in children appears to be less grave, and asymptomatic and mild cases have been reported in the range between 35 and 56% (9).

The incubation period ranges from 2 to 14 days, with the greatest frequency in a range between 3 and 7 days (10), as the virus can survive in the environment as long as 9 days. Common disinfectants (sodium hypochlorite) are effective against the virus. Transmission by asymptomatic subjects has not been excluded, and thus currently isolation is the first form of prevention (11).

Notwithstanding this information, the infection from COVID-19 has been faced as an epidemic, through measures to enforce a high degree of isolation. This has entailed a complex management model with individual measures, structures and logistics aimed at avoiding the spread of the infection in the nosocomial sphere and consequently in the community.

## The Clinical Aspects of Covid 2019 in Children

Globally, respiratory viruses are among the principal causes of death and morbidity in minors (12). Early in the outbreak, the National Respiratory Diseases Clinical Research Center and Respiratory Diseases Group of the Chinese Pediatric Association began a study on the clinical features of COVID 19 in pediatric patients, providing the first information on the characteristics of COVID 19 flu in children (13), which currently seems to be less severe than the clinical forms in adults (14).

One Chinese study indicated that children are seldom attacked by COVID 19 or SARS-CoV, and suggested that this was due to the Chinese vaccination program for minors. In particular, “the RNA-virus vaccines and the adjuvants in vaccine programs may help children escape from the infection” (15). However, according to other authors, the number of pediatric patients suffering from COVID 19 “could rise in the future, and a lower number of pediatric patients at the beginning of a pandemic does not necessarily mean that children are less susceptible to the infection” (16, 17). Complete understanding of the physiological mechanisms underlying the lower rate of infection among children could help in battling the diametrically opposite gravity of the infection in adults.

Detailed study of the genetic characteristics of patients is needed to explain the extreme variability of clinical phenotypes as well as the variation in responsiveness to certain drugs (18, 19).

The GEFACOVID project will develop a blood test that will serve to “stratify patients based on genetic traits influencing the response to coronavirus infection, and, in turn, choose the most suitable treatment approach” (20).

On the basis of experiences reported in China (5), the clinical manifestations of the COVID 19 infection in children are “fever, fatigue and cough, nasal congestion, runny nose, expectoration, diarrhea, headache, etc.” The fever is generally low or absent. After about a week of infection, children may develop “dyspnea, cyanosis accompanied by systemic toxic symptoms, such as malaise or restlessness, poor feeding, bad appetite and less activity.” There can also be “abdominal discomfort, vomiting, abdominal pain and diarrhea.”

Respiratory insufficiency may arise, but can easily be corrected with a few days of oxygen therapy administered by nasal catheter or mask. In the most severe cases, “septic shock, metabolic acidosis and irreversible bleeding and coagulation dysfunction may occur.” “In the early phase of the disease, white blood cell count is normal or decreased, or with decreased lymphocyte count (21).”

Authors report the presence of different features of the MIS-C (e.g., toxic shock syndrome, secondary hemophagocytic lymphohistiocytosis, or macrophage activation syndrome) (22) and Kawasaki-like disease (23).

Radiological signs attest the presence of viral pneumonia at the beginning, which can advance to the point of signs of lung consolidation (24, 25).

Lung ultrasound abnormalities have been described, so an ultrasound approach could represent an adjunct tool for achieving a rapid severity assessment of COVID-19 lung involvement for children population (9).

There has been no indication of vertical transmission from mother to infant, not even through maternal milk (25). In fact, the Italian Society of Neonatology has proposed that a woman who has tested positive for corona virus but is asymptomatic should be allowed to nurse and be close to her newborn. Instead, if she shows symptoms of fever, coughing and respiratory secretions, she and her newborn should be separated, if she consents to this and if the logistics of the hospital permit it (26).

No standard treatment has been identified, and current treatment plans for children are adapted from those for adults (20).

Contact between medical personnel and patients must be kept to the minimum; visitors should have limited access, and wear proper protective items (5). In such a context, there is also need for psychological support (25, 27). In fact, some studied have also asked whether a few weeks or months of forced separation can have enduring effects, and noted that brief traumatic events can have life-long consequences (28, 29), such as posttraumatic stress disorder, anxiety disorder, depression, aggression, psychosomatic complaint and suicidal ideation (30, 31).

## Italian Care for Minor Patients

The choice in Chinese healthcare protocols to isolate minors suffering from COVID 19 and to limit their contact with others seems quite problematic for an Italian setting. In general, visitors who provide assistance to patients have been identified as the source of various infections, including flu and SARS, but no studies have been conducted on effective methods for screening the health status of visitors (12). Similarly, no proof has been provided for the efficacy of having visitors wear personal protection devices, such as masks, gloves, protective eyewear, or gowns. Further, if these barrier precautions are not used correctly, those who are in very close contact with patients, supporting and feeding them, could then come into contact with other patients and transmit the disease to them.

The situation in which a healthy parent desires to stay with a child sick with COVID 19 presents complex questions. Italian guidelines on precautionary isolation contain no explicit exceptions when the patient is a minor. However, in such a case, even if the parent uses personal protection devices to prevent contagion, allowing him or her to stay there may indicate a lack of proper attention to and protection of the health of the parent.

Italian legislation recognizes the minor as a vulnerable subject for whom specific protections should be provided by parents or legal representatives, who are also required by law to care for the minor's health. In the case of disagreement between parents, a judge may assign decisional power to the parent who in the particular case is deemed most suitable for pursuing the interests of the child. In addition, the judge can void parental authority when the parent violates or neglects responsibilities or abuses this power with grave prejudice to the child (art. 330 Civil Code) (32).

Minors “must receive information about healthcare choices in a form appropriate to their ability to understand, so that they can express their wishes” (L. 219/17 art. 3).

Informed consent to treatment for a minor is provided by parents or legal guardians (33), according to Law n. 219 of December 22, 2017 “*Regulations on informed consent and advance directives for treatment*,” which establishes the limits and methods for acquiring consent (34).

When off label treatment is proposed, as may be the case with COVID 19, the weight of the parents' informed consent is even more important (35). In these cases, if physicians fail to give parents scientific evidence in support of the need for such treatment, parents may oppose them. Clearly, the COVID 19 epidemic is a situation of emergency and experimentation. Article 5 of Law n. 219 on *Shared planning of treatment* calls for more adequate counseling and for the involvement of parents, the minor, and the physician in treatment choices, but this does not seems completely adequate for managing situations of conflict that may arise among them, especially when the treatment proposed is not backed by sufficient scientific evidence. Can an emergency situation justify the administration of drugs of uncertain efficacy, especially when the patient is a minor? In addition, since the core issue is essentially clinical experimentation, would it not be useful to request urgently the opinion of an Ethics Committee? Would it not be desirable to receive some direction from the Health Ministry? This would be valuable not only for minors, but for all gravely ill subjects who are incapable of expressing their own decision.

Fortunately, COVID 19 has not generated dramatic clinical manifestations in minors, and Italy has not had to face these problematic issues in the management of pediatric patients.

Parri et al. believe that “the management of pediatric COVID-19 patients in the Emergency Department is represented by the organizational burden (e.g., management of patient flow), rather than any one specific clinical task,” so proper allocation of resources and treatment is needed, as well as strong collaboration at different levels of the health care system (9).

Other considerations not strictly of a healthcare nature concern the right to study.

It is evident that the restrictive regulations introduced with the DPCM of March 4 2020 (36) closing all schools and universities in Italy will undermine the right to study of minors, but it was ordered with the intent of preventing possible COVID 19 infection, which in the long run may be the greater good.

Subsequent DPCMs confirmed the suspension of teaching in presence until 31 July 2020.

At the same time, distance learning was activated to safeguard the right to study, and special arrangements were made for in-person lessons for children with disabilities (37).

The Ministerial Decree of 26 March 2020, n. 186 (38) stipulated that, throughout the period of suspension of educational activities, local authorities could assist pupils with disabilities through the provision of individual household benefits, aimed in particular at supporting the use of distance learning activities.

Ministerial Decree n. 39 of 26 June 2020 (39) adopted the Document for the planning of school, educational and training activities in all institutions of the national education system for the school year 2020/2021.

With Ministerial Decree n. 80 of 3 August 2020 (40), the Guideline and Guidance Document was adopted for the resumption of in presence activities at educational services and children's schools.

On August 6, 2020 (41) the Security Protocol ordered the start of the school year in compliance with safety rules provided by Ministerial Decree 39/2020 (39).

The actions outlined in these documents are based on a plausible setting for the first half of the year of the next school year (2020/2021) assuming the prolongation of the pandemic at a global level and new episodes of contagion at a local level in autumn-winter, up to a more disastrous perspective regarding a new temporary suspension of teaching activities.

Many authors have reported that minors indirectly suffered social health consequences from the restrictions established to limit the spread of pandemic, such as social distancing measures, school closures and the cessation of recreational activities, which are important for the cultural, social and psychological growth of children and adolescents (42–44).

Buonsenso et al. believe that the routine and long-lasting separation of children from their families, lengthy school closures and not being able to visit public outdoor spaces or play with other children have affected the mental health of children and are no longer ethically acceptable (27).

In fact, they believe that over prolonged periods, schools closures are more damaging to children and to society in the long term than risks of transmission of COVID-19 (45).

## Conclusion

The current Italian legislation does not allow for concrete and active participation of minors, especially those under the age of 12, in the discussion of choices about their health, even when the minor shows good capacity of discernment (46).

The healthcare system and society should guarantee the well-being of minors by reflecting upon their point of view, discussing it coherently, and analyzing the various aspects to reach well-thought-out solutions in the best interests of the children/adolescents.

Since the beginning of the pandemic emergency, minors have been labeled as vectors for the transmission of SARS-CoV-2, and the measures of social and family isolation have turned their world upside down. Even though studies have suggested that children and adolescents have lower susceptibility to the virus than adults and play a lesser role in transmission (47–49), activities that serve this age group have been the first to be strongly limited.

Recently, the National Committee for Bioethics (NBC) urged that possible measures to counter the pandemic should be evaluated according to the ethical and juridical criterion of the best interests of minors, and those whose negative consequences most affect this age group should be limited as much as possible (50).

The NBC called for the promotion of consciousness-raising about the responsibility to care for individual and public health, particularly regarding needs related to the pandemic, as a preferable alternative to limitation of activities dedicated to minors. This effort should be part of the school curriculum and should also involve extracurricular educational initiatives that include family members as well.

The concrete risk of having an entire generation of children and adolescents with psychological problems brought on by pandemic-related restrictions, even when this age group shows a fairly minimal clinical expression of COVID-19, should give pause to institutions; they should take into consideration the bioethical issues that are inevitably interwoven with political, economic, social and healthcare ones.

## Data Availability Statement

The original contributions presented in the study are included in the article/supplementary material, further inquiries can be directed to the corresponding author/s.

## Author Contributions

RS and MC: substantial contributions to the conception or design of the work or the acquisition of information. PF and NC: drafting the work or revising it critically for important intellectual content. All authors read and approved the final manuscript.

## Conflict of Interest

The authors declare that the research was conducted in the absence of any commercial or financial relationships that could be construed as a potential conflict of interest.
